# Stochastic binary synapses having sigmoidal cumulative distribution functions for unsupervised learning with spike timing-dependent plasticity

**DOI:** 10.1038/s41598-021-97583-y

**Published:** 2021-09-14

**Authors:** Yoshifumi Nishi, Kumiko Nomura, Takao Marukame, Koichi Mizushima

**Affiliations:** Frontier Research Laboratory, Corporate R&D Center, Toshiba Corporation, 1, Komukai-Toshiba-Cho, Saiwai-ku, Kawasaki, 212-8582 Japan

**Keywords:** Electrical and electronic engineering, Electronic devices, Computational neuroscience

## Abstract

Spike timing-dependent plasticity (STDP), which is widely studied as a fundamental synaptic update rule for neuromorphic hardware, requires precise control of continuous weights. From the viewpoint of hardware implementation, a simplified update rule is desirable. Although simplified STDP with stochastic binary synapses was proposed previously, we find that it leads to degradation of memory maintenance during learning, which is unfavourable for unsupervised online learning. In this work, we propose a stochastic binary synaptic model where the cumulative probability of the weight change evolves in a sigmoidal fashion with potentiation or depression trials, which can be implemented using a pair of switching devices consisting of serially connected multiple binary memristors. As a benchmark test we perform simulations of unsupervised learning of MNIST images with a two-layer network and show that simplified STDP in combination with this model can outperform conventional rules with continuous weights not only in memory maintenance but also in recognition accuracy. Our method achieves 97.3% in recognition accuracy, which is higher than that reported with standard STDP in the same framework. We also show that the high performance of our learning rule is robust against device-to-device variability of the memristor's probabilistic behaviour.

## Introduction

Spike timing-dependent plasticity (STDP), which was discovered in biological neuronal systems^1–3^, has established its position as the most fundamental synaptic update rule also in artificial neuromorphic hardware where spiking neural networks (SNNs) are implemented to mimic the information processing principle of the biological brain^4–8^. Many attempts have been made to implement STDP in SNN hardware systems in order to realise autonomous online learning which our brains are always doing with an ultimately low power of 20 W.

STDP is a synaptic update rule where a synaptic weight is depressed when a pre-synaptic spikes comes after the post-synaptic neuron fires, and potentiated when the post-synaptic neuron fires after an arrival of a pre-synaptic spike. In standard STDP models, the amount of the weight change depends on ${t}_{pre}-{t}_{post}$ exponentially, where ${t}_{post}$ and ${t}_{pre}$ denote the time at which the post-neuron fires and the time at which a pre-synaptic spike arrives, respectively^9^. Thus, to implement STDP in hardware straightforwardly, we need multi-bit memories to store synaptic weights with high precision, computing units to calculate the weight changes, and memory controllers to update the memories. However, such hardware-heavy implementation would be unfavourable for area-efficient and low power neuromorphic chips. It is desirable to employ devices that can reproduce STDP operation with as few hardware components as possible.

One of the most studied devices for STDP implementation is the memristor^9–17^. A memristor is a two-terminal passive device whose resistance changes in accordance with the polarity, amplitude and duration of the applied voltage^10^. The variable conductance of a memristor can represent the plastic weight of a synapse. Simultaneous application of specifically-shaped voltage pulses to both terminals updates the resistance depending on the timing of the two pulses, resulting in STDP-like behaviour^9^. Controlling the resistances of a population of memristors in an analogue fashion, however, is not an easy task. Because of the uncontrollable variability, careful pulse tuning is required for each device to reproduce its designed behaviour^18^, hindering practical use of memristors for synaptic devices.

A solution to avoid such difficulty is to use memristors in a binary fashion^19,20^. Several types of oxide-based memristors show binary behaviour after a forming process^21–24^. When a voltage with one polarity is applied to a memristor, its resistance state undergoes a SET transition from a high-resistance state (HRS) to a low-resistance state (LRS). A voltage with the opposite polarity arises a RESET transition from an LRS to an HRS^25^. Thus we need only consider whether the device is in an LRS or an HRS, and we do not need to consider the precise analogue resistance of individual devices.

A problem here is that synaptic weights are assumed to be continuous in standard STDP models. In fact, it is widely known that, in general, continuous or multi-bit weights are required for learning in neural networks, although inference tasks can be performed with binary synapses^26^. For learning in neural networks with binary synapses, auxiliary continuous variables may be added instead of using continuous synaptic weights. For example, a learning algorithm proposed by Brader, et al. employs binary synapses, but each synapse has a dynamic continuous variable by which the weight, 0 or 1, is determined^27^. Thus, this approach does not reduce the difficulty of controlling the continuous weights because the new variables merely take the place of the synaptic weights; another hardware mechanism to replace the auxiliary continuous variables is required.

Stochastic operation of binary synapses is one possible solution^28^. Suri, et al. showed that the probabilistic SET and RESET of binary memristors can be used for STDP-based learning^19^. In their learning rule, which we refer to as stochastic simplified STDP (stochastic S-STDP), the information of spike-timing is encoded as a probability of SET or RESET instead of continuous increment or decrement of the weight. The stochasticity of the switching behaviour originates from the randomness of the configuration of the ions or vacancies in the insulating film in a memristor^21,29,30^. Despite its simplicity, the learning performance of stochastic S-STDP is not as high as that of a deterministic rule with continuous weights, as shown in the following section.

Weight change in standard STDP models generally depends not only on ${t}_{pre}-{t}_{post}$ but also on the present weight value of the target synapse^9,13,31–36^. This is originated from the fact that the dynamic range of a synaptic weight is not unlimited and has its upper and lower bounds. When a synapse having a weight close to the upper bound is potentiated, the increment should be small enough so that the resulting weight value does not exceed the upper bound. Similarly, depression should be small enough if the present value is close to the lower bound. In this way, the amount of the weight update should be given depending on the present weight. Since weight-dependence in a synaptic update rule is an effective factor for the performance and stability of learning^9,33^, it should be considered carefully in designing a learning system for neuromorphic hardware. Asymmetric linear dependence, where synaptic weight change is proportional to a linear function of the present weight (hence, the weight increases or decreases exponentially as a function of the number of potentiation or depression), is widely known as a simple model. However, Park et al. proposed that a symmetric model where the weight changes with the potentiation or depression operations in a sigmoidal fashion improves the memory maintenance of the network^37^.

For a binary synaptic system, however, weight-dependence does not make sense because a weight is always 0 or 1. Our approach in this work is to focus on the expected value of a weight rather than the actual value. To control the expected value in a sigmoidal fashion with potentiation (depression) trials, the probability of switching from 0 to 1 (from 1 to 0) cannot be a constant and must be dependent on the number of trials. A question arising from this scenario is how to control the probability in accordance with the operation cycle. It would be area-expensive to implement a control system in a semiconductor chip. In this work we propose the use of a stochastic switch consisting of serially connected multiple binary memristors. The stochastic nature of such a switch can be described by a gamma distribution, which leads to a sigmoidal dependence of the expected weight on the number of trials. Using Brian Simulator^38^, we show that our stochastic learning rule improves the performance in MNIST image learning tasks in a two-layer SNN.

## Results

### Expected weights in stochastic S-STDP

**Figure 1:**
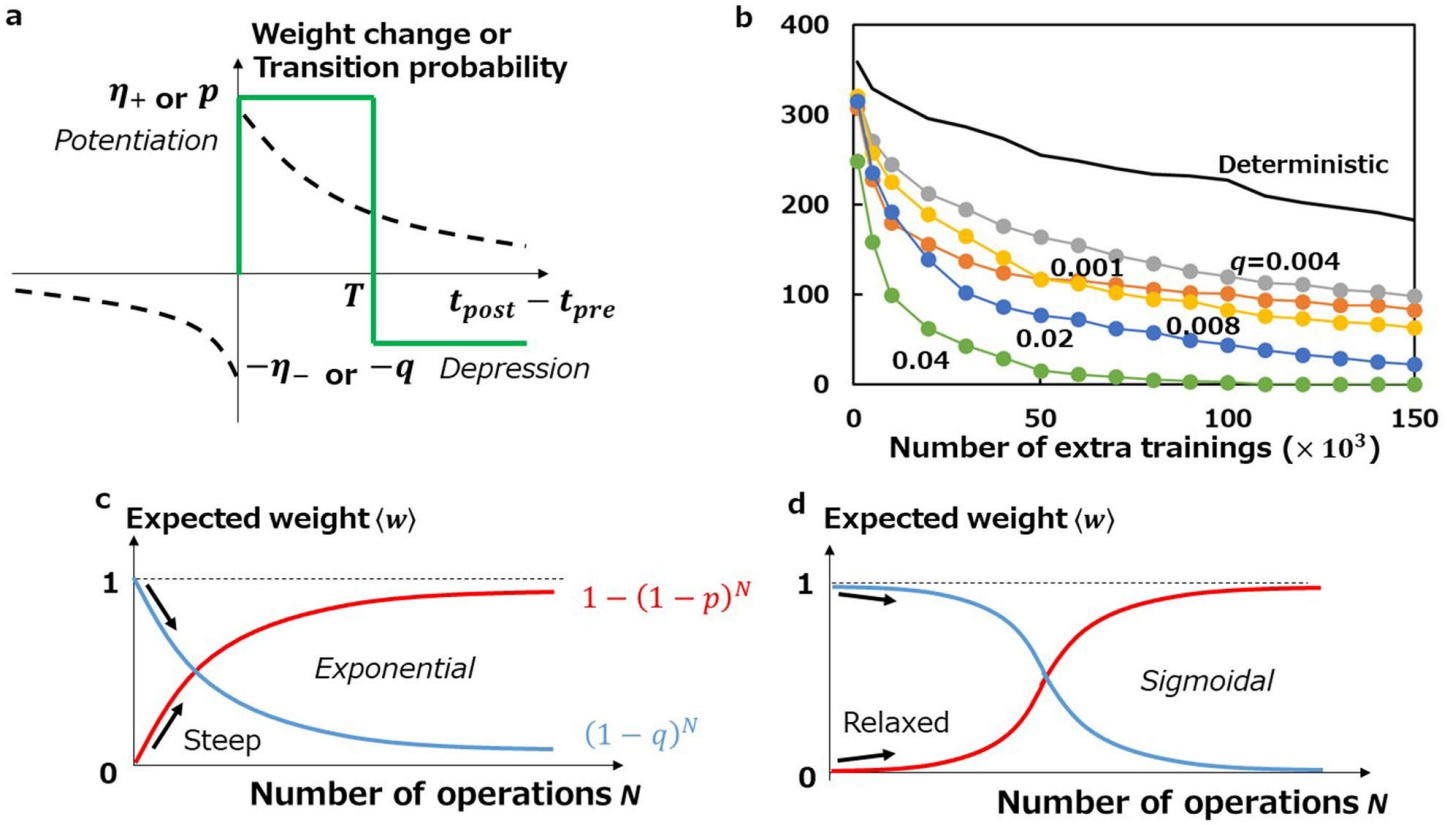
Introduction of stochastic S-STDP. (**a)** Schematics of S-STDP. While the synaptic weight update has exponential-like dependence on ${t}_{post}-{t}_{pre}$ in standard STDP (broken curves), S-STDP is characterised by a rectangular dependence (green line). Since an update takes place at the occurrence of a post-neuron's fire, we only consider the case of ${t}_{post}-{t}_{pre}>0$ for S-STDP. In the scheme of stochastic S-STDP with binary weights, the weight change ${\eta }_{+}$ and ${\eta }_{-}$ are read as the transition probabilities $p$ from $w=0$ to $1$ for potentiation and $q$ from $1$ to $0$ for depression, respectively. (**b)** Memory maintenance characteristics of learning with conventional stochastic S-STDP. The number of neurons that retain the digit memorised during the initial learning is plotted against the number of extra trainings (number of training samples presented for additional learning). Depression probability $q$ is varied while potentiation $p$ is fixed at 0.04. For comparison the characteristic of learning with deterministic S-STDP using continuous weights is shown as a thick line, for which we employ a linear weight-dependent update model^33,37^, where $\Delta w={\eta }_{+}\left({w}_{max}-w\right)$ for potentiation and $\Delta w={\eta }_{-}\left(w-{w}_{min}\right)$ for depression with ${\eta }_{+}=0.04$ and ${\eta }_{-}=0.008$, respectively. (**c,d)** Evolutions of expected weights for conventional stochastic S-STDP **(c)** and proposed "sigmoidal stochastic" S-STDP **(d)**.

For continuous synapses, deterministic simplified STDP (deterministic S-STDP) can be employed. Deterministic S-STDP increments or decrements a weight by a small amount ${\eta }_{+}$ or ${\eta }_{-}$, respectively, at the moment of a post-neuron's firing depending on whether $0<{t}_{post}-{t}_{pre}<T$ or not^35^. In contrast, stochastic S-STDP potentiates a binary synapse from 0 to 1 with a probability $p$ in the former case, or depresses from 1 to 0 with a probability $q$ in the latter case (Fig. 1a). It is so simple that precise control of the memristive resistance is unnecessary. Instead it requires binary memristors to perform switching with a fixed probability ($p$ or $q$) regardless of the operation history. It should be noted that this requirement is not trivial because the stochastic properties of a memristor may vary from cycle to cycle owing microscopic internal change due to Joule heating^30,39^. Fortunately, however, it is possible to find a voltage pulse condition with which the SET probability is kept constant independently of the voltage application history^30^. We refer to stochastic S-STDP with constant probabilities as conventional stochastic S-STDP.

In the scheme of S-STDP, both deterministic and stochastic, when a neuron fires, only the synapses that have received a spike within a period $T$ prior to the fire are potentiated and all the other synapses afferent to the neuron are depressed. Consequently, the sum of the synaptic weights afferent to the neuron is kept around a constant, depending on the balance of potentiation and depression^35^. This means that synaptic normalization or synaptic scaling mechanism, which keeps the weight-sum at a constant and works as a homeostatic mechanism for the stability of the weight distribution^34,40–43^, is inherent in this rule (see also the Discussion section).

To discuss the performance of synaptic update rules, we perform simulations of unsupervised learning of MNIST images in a two-layer SNN^34,44,45^ and evaluate recognition accuracy and memory maintenance (see the Methods section for details). Note that unsupervised learning in a two-layer SNN is studied as a basic model of Bayesian computation in cortical microcircuits^45,46^. Selecting $p$ and $q$ appropriately, we can observe relatively high accuracy for conventional stochastic S-STDP. Our best accuracy 85.5% is achieved with $p=0.04$ and $q=0.008$ (Supplementary Note 1). Note that our interest is in benchmarking the performance of update rules as unsupervised learning algorithms in SNNs, not in the recognition task itself, much less in achieving higher accuracy than other machine learning methods.

Memory maintenance, which is represented by the number of neurons holding the initial memory after extra trainings, is evaluated to argue the stability of learning. In general, the number of neurons holding the initial memory decreases with the number of extra trainings because neurons change their memory when trained with new samples. From the viewpoint of online learning applications, the decay should be slow enough that the network can retain its memory and work from its long term experience. In Fig. 1b, all the curves obtained with conventional stochastic S-STDP, including the curve achieving the best accuracy with $p=0.04$ and $q=0.008$, are much lower than those obtained with deterministic S-STDP. It is desirable that those curves lie at higher positions with smaller decay rates.

To discuss how to improve the memory maintenance of the network with stochastic S-STDP, we focus on the behaviour of expected values of weights. Let us consider a binary synapse with weight $w=0$ and assume that a probabilistic potentiation is repeated $N$ times. Then, the cumulative probability of $w=1$ (i.e., the probability of finding $w=1$), $P\left(N\right)$, and the expected weight $\langle w\rangle$ can be expressed as

1 $$P\left(N\right)=1-{\left(1-p\right)}^{N}=1-\mathrm{exp}\left(-\lambda N\right), \langle w\rangle =0+1\times P\left(N\right)=1-\mathrm{exp}\left(-\lambda N\right),$$

where $\lambda =-\mathrm{ln}\left(1-p\right)\approx p$ (for $p\ll 1$). Similarly, for depression from $w=1$ to 0, the cumulative probability $Q\left(N\right)$ and the expected weight $\langle w\rangle$ can be expressed as

2 $$Q\left(N\right)=1-{\left(1-q\right)}^{N}=1-\mathrm{exp}\left(-\mu N\right), \langle w\rangle =1-1\times Q\left(N\right)=\mathrm{exp}\left(-\mu N\right),$$

where $\mu =-\mathrm{ln}\left(1-q\right)\approx q$ (for $q\ll 1$). As can be seen from Eqs. (1) and (2), $\langle w\rangle$ is written as an exponential function of $N$. Note that this corresponds to a linear weight-dependent update of the continuous weight^37^. Because an exponential evolution starts with a steep rise (Fig. 1c), the dominant change of $\langle w\rangle$ takes place at an early stage in a series of potentiation or depression stimuli. We hypothesise that such steepness is a cause of the memory instability. To improve the memory maintenance, we propose that the evolution of $\langle w\rangle$ should start with a gentle rise and be accelerated gradually after a certain period of stimulus (Fig. 1d); in other words, $\langle w\rangle$ should be sigmoidal with $N$ rather than exponential.

### Realisation of sigmoidal evolutions of expected weights

If an expected weight $\langle w\rangle$ evolves in a sigmoidal fashion with $N$, the potentiation and depression probabilities cannot be constant; they must start with small values and become larger with *N*. It would be area-expensive, and thus hardware-unfriendly, to prepare elaborate circuits to control the probabilities in accordance with *N*. For hardware implementation, it is desirable to exploit a random phenomenon whose occurrence probability increases with the number of trials automatically.

**Figure 2:**
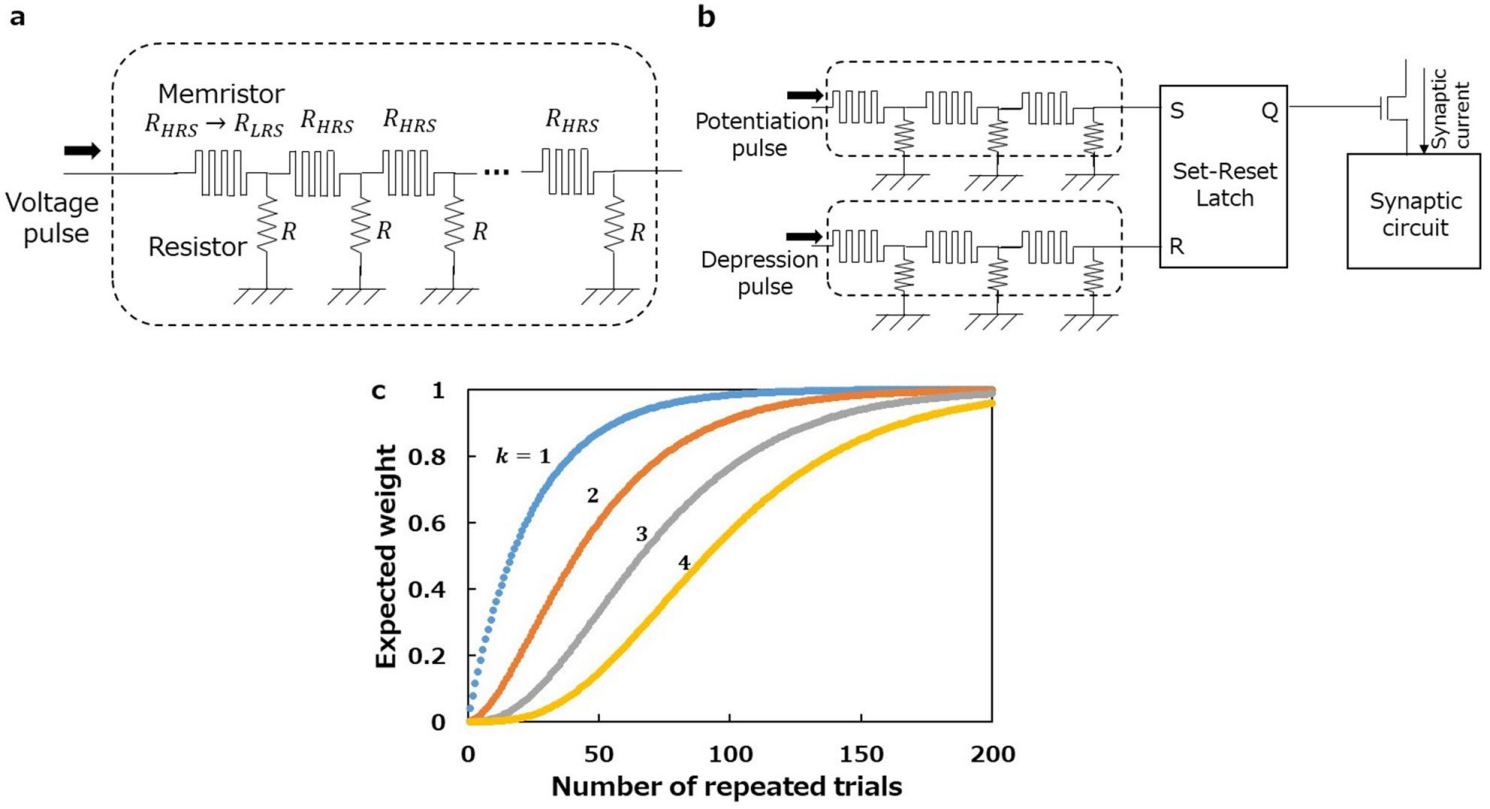
Switching device having serially connected binary memristors to realise the sigmoidal cumulative probability. (**a)** Schematics of the device. Connection nodes between two memristors are grounded through a resistor. The resistance $R$ is selected to be sufficiently larger than the LRS resistance (${R}_{LRS}$) and sufficiently smaller than the HRS resistance (${R}_{HRS}$); ${R}_{LRS}\ll R\ll {R}_{HRS}$. This is possible because the ratio of ${R}_{HRS}$ to ${R}_{LRS}$ is several orders of magnitude in binary memristors (even if integrated with CMOS transistors^47^). Voltage pulses are applied to the left terminal, inducing SETs in memristors serially from left to right. To initialise the device, all the memristors should be RESET in parallel in a deterministic fashion. Therefore, a switching device should be designed with multiplexers so that each memristor can be accessed independently for RESET; for clarity, these are not illustrated here. (**b)** Schematics of the binary synapse using a pair of switching devices of serially connected binary memristors. If the upper device is open, the output of the latch Q is high. Then the synaptic current flows, corresponding to $w=1$. Conversely, if the lower is open, the synaptic current does not flow, corresponding to $w=0$. See Supplementary Note 2) for detailed operations. **c** Expected weight curves calculated using Eq. (6), where $\lambda =0.04$.

The solution we propose here is to exploit serially connected stochastic switching elements as a random event source. Let us consider the switching device shown in Fig. 2a, which consists of $k$ binary memristors connected in series. We assume that those memristors have exponential SET time statistics and thus a SET can be regarded as a Poissonian random event with a constant probability given by

3 $$p=1-\mathrm{exp}\left(-\frac{\Delta t}{\tau }\right),$$

where $\Delta t$ is the width of voltage pulses applied to the memristors and $\tau$ is a constant. This multiple memristor-switching device is conductive (in the ON state) only when all the memristors are in an LRS. Otherwise, it is non-conductive (in the OFF state) because at least one memristor is in an HRS, and is therefore insulating. We initialise the device by RESETting all the memristors in an HRS and then apply voltage pulses to the left terminal. In this situation, only the leftmost memristor receives the effective voltage. Applying pulses several times eventually gives rise to a SET in the leftmost memristor. Once the leftmost memristor switches to an LRS, voltage pulses can go through it and reach the next memristor, inducing a SET there. In this way, memristors in the switching device undergo SETs stochastically one after another from left to right. Finally, the voltage pulses SET the rightmost memristor to an LRS, turning the whole switching device to the ON state.

Let $x$ be the cumulataive duration of voltage pulses applied until the device switches from OFF to ON. Then, the number of applied pulses is written as $N=\llcorner x/\Delta t \lrcorner+1$, where $\llcorner a \lrcorner$ is the largest integer that does not exceed $a$. The probability of a switching event from OFF to ON (i.e., occurrence of a SET in the $k$-th memristor) between $x$ and $x+dx$ is given by ${p}_{k}\left(x\right)dx$, where ${p}_{k}\left(x\right)$ is a probability distribution function of a gamma distribution with a shape parameter $k$ and a mean parameter $1/\tau$ as

4 $${p}_{k}\left(x\right)=\frac{{x}^{k-1}}{\Gamma \left(k\right){\tau }^{k}}\mathrm{exp}\left(-\frac{x}{\tau }\right).$$

The cumulative probability ${P}_{k}\left(N\right)$, that is, the probability of finding the switching device in the ON sate after applying $N$ pulses is given by

5 $${P}_{k}\left(N\right)={\int }_{0}^{\left(N-1\right)\Delta t}{p}_{k}\left(x\right)dx\approx \frac{1}{\Gamma \left(k\right){\tau }^{k}}\sum_{n=0}^{N-1}{\left(n\Delta t\right)}^{k-1}\mathrm{exp}\left(-\frac{n\Delta t}{\tau }\right)\Delta t=\frac{{\lambda }^{k}}{\left(k-1\right)!}\sum_{n=0}^{N-1}{n}^{k-1}\mathrm{exp}\left(-\lambda n\right),$$

where $\lambda =\Delta t/\tau =-\mathrm{ln}\left(1-p\right)$. By setting $k=1$, Eq. (5) is reduced to Eq. (1) as ${P}_{1}\left(N\right)={\sum }_{n}\lambda {e}^{-\lambda n}\approx 1-\mathrm{exp}\left(-\lambda N\right)$ for $\lambda \ll 1$. This indicates that Eq. (5) can be understood as a generalisation of Eq. (1) and gives us justification to use the notations of $\lambda$ and $p$ in common with Eq. (1).

Using a pair of the switching devices discussed above in combination with a synaptic circuit^48^ makes it possible to build a binary synaptic device where potentiation from $w=0$ to $1$ is represented by a switching operation of one device, and depression from $w=1$ to $0$ is represented by the operation of the other device as shown in Fig. 2b (see Supplementary Note 2 for detailed information). In the case of potentiation, $\langle w\rangle$ evolves with $N$ as

6 $$\langle w\rangle =0+1\times {P}_{k}\left(N\right)=\frac{{\lambda }^{k}}{\left(k-1\right)!}\sum_{n=0}^{N-1}{n}^{k-1}\mathrm{exp}\left(-\lambda n\right).$$

Calculating $\langle w\rangle$ evolutions for $k=1, 2, 3$ and $4$, sigmoidal $N$ dependence is observed for $k\ge 2$, while $k=1$ gives an exponential dependence (Fig. 2c). The curve is more relaxed for larger $k$; in other words, the larger $k$ is, the more pulses are required to SET all $k$ memristors.

The depression proceeds in exactly the same way. For discrimination between the first and the second switching devices in a synapse, we use ${Q}_{k}\left(N\right)$ , ${q}_{k}\left(N\right)$ and $q$ for the second switching device to denote the cumulative probability, the switching probability at the $N$-th depression and the switching probability of the memristors. Then $\langle w\rangle$ for depression is given by

7 $$\langle w\rangle =1-1\times {Q}_{k}\left(N\right)=1-\frac{{\mu }^{k}}{\left(k-1\right)!}\sum_{n=0}^{N-1}{n}^{k-1}\mathrm{exp}\left(-\mu n\right),$$

where $\mu =-\mathrm{ln}\left(1-q\right)$ is a parameter corresponding to $\lambda$ in Eq. (6).

### Learning with sigmoidal stochastic S-STDP

**Figure 3:**
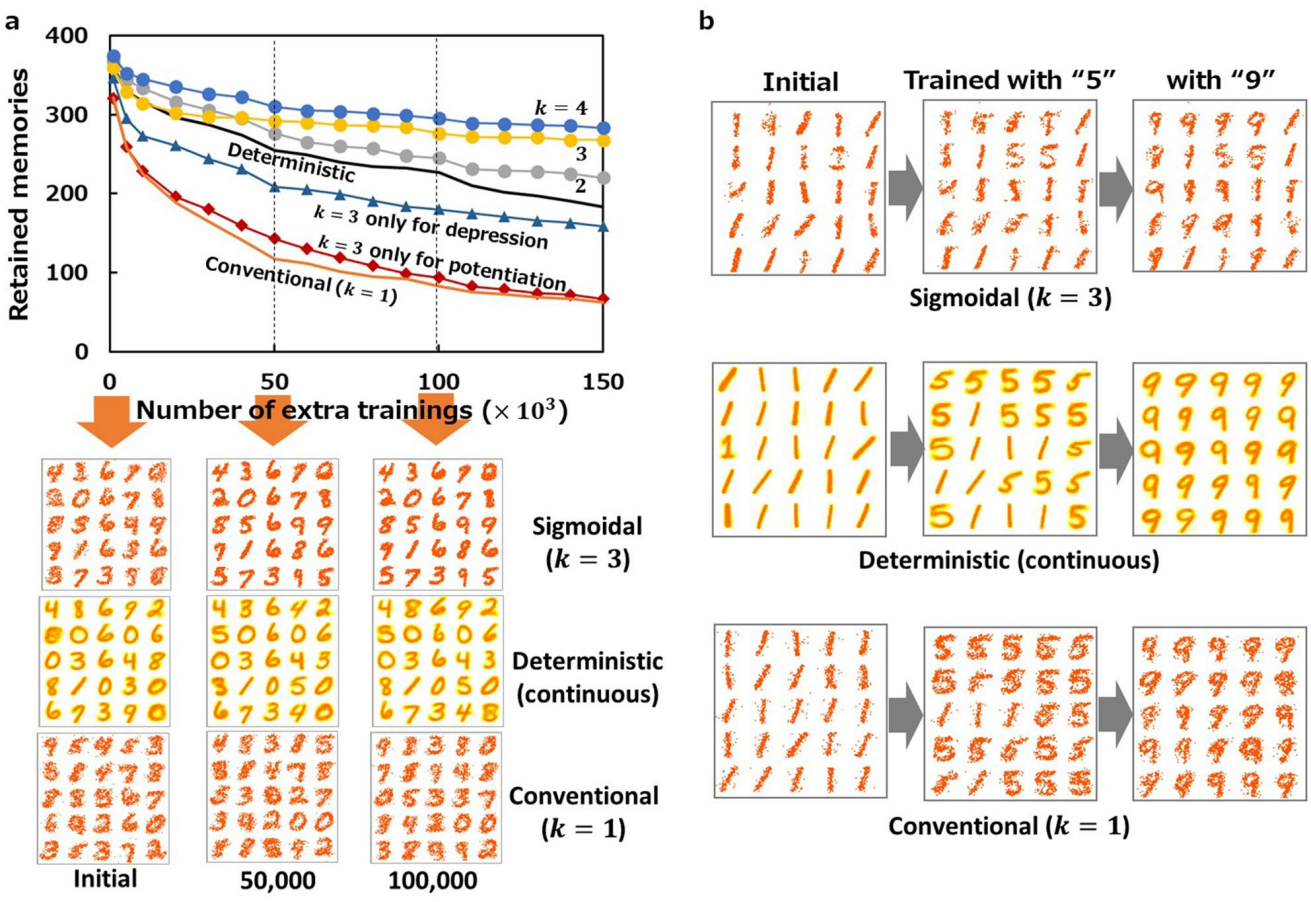
Memory maintenance characteristics of learning with sigmoidal stochastic S-STDP. (**a)** Memory maintenance curves obtained with sigmoidal stochastic S-STDP in the cases of $k= 2$ and $3$ (with $p=0.13$ and $q=0.03$) and $k=4$ (with $p=0.2$ and $q=0.08$). For comparison, those obtained with conventional ($k=1$) and deterministic S-STDP are shown (taken from Fig. 1b). Furthermore, the memory maintenance curves in the case where the sigmoidal rule of $k=3$ with $p=0.13$ is applied only for potentiation (depression is performed with the conventional rule with $q=0.008$), and in the opposite case (with $p=0.04$ and $q=0.03$) are also plotted. Colour intensity maps of synaptic weights afferent to $5\times 5=25$ excitatory neurons out of 400 in the second layer are shown after the initial training and 50,000 and 100,000 additional trainings. Each pixel in the map correspond to a weight. Thus, each panel contains $28\times 28\times 25=\mathrm{19,600}$ pixels. White and red indicate 0 and 1, respectively. The intermediate values for continuous weights are represented by yellowish colours. **(b)** Colour intensity maps showing the results of the benchmark test of memory maintenance (described in the text).

Simulation results support our hypothesis that learning with S-STDP using a stochastic sigmoidal switching model discussed above (hereafter referred to as sigmoidal stochastic S-STDP) improves the memory maintenance (Fig. 3a). In the simulation, we employ an update algorithm that follows the stochastic behaviour of a pair of multiple memristor-devices. The algorithm is explained in the Methods section. Even in the case of the smallest $k$ (i.e. $k=2$) the maintenance curve is clearly higher than that obtained with deterministic S-STDP. Excellent improvements are observed for $k=3$ and $4$, where $\langle w\rangle$ evolution curves definitely have sigmoid shapes (see Fig. 2c). Employing a sigmoidal rule only for potentiation and leaving the depression conventional does not improve the memory maintenance. Some improvement is observed in the opposite case (i.e., sigmoidal for depression and conventional for potentiation), but the retention is still worse than that obtained with the deterministic rule. Note that for all the experiments in this work, we tried various combination of $p$ and $q$ under each condition and picked up the best one for fair comparison among several conditions.

Colour intensity maps of the plastic synaptic weights provide us with visual insights into the neurons' memory behaviour. Here weight maps of 25 excitatory neurons (out of 400) are shown. Each of $5\times 5$ patterns consisting of $28\times 28$ pixels corresponds to a neuron's memory. The memorised patterns become sharper but do not show drastic change during additional learning in the case of sigmoidal stochastic S-STDP. In contrast, more than half of the neurons alter their patterns after being presented with new samples in the conventional case. The deterministic case is in-between: some of the initial patterns are rewritten to other similar patterns ('8' to '3', '3' to '5', '9' to '4', and '0' to '8') during training. It is interesting to see that weight maps of deterministic S-STDP appear vivid to the human eye. Patterns with binary weights appear somewhat dull, but this does not necessarily lead to degradation of recognition performance, as shown below.

To demonstrate the memory maintenance further, we perform another experiment. We first initialise the network by presenting 6,000 images of the digit '1'. After initialization, the network is trained with 3000 images of '5', followed by further training with 3,000 images of '9'. Weight maps after each phase are compared in Fig. 3b. Whereas all the neurons' memories are overwritten with '9' after training with '9' in both the deterministic and conventional cases, patterns of '1' and '5' coexist with those of '9' in the sigmoidal case. This result indicates that the robustness of a neuron's memory increases when trained with sigmoidal stochastic S-STDP.

**Figure 4:**
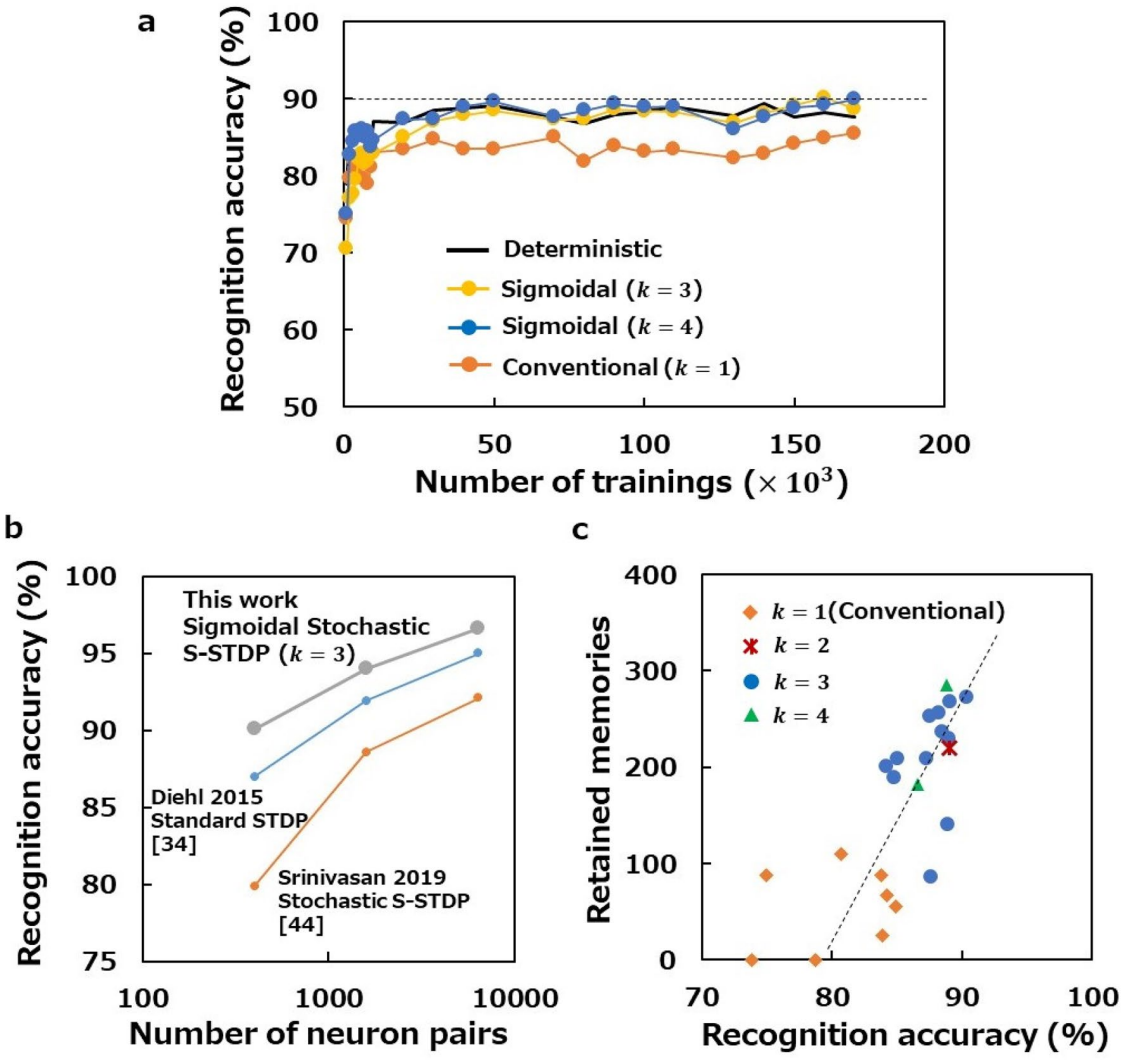
Recognition accuracy. (**a)** Evolutions of recognition accuracy obtained using sigmoidal stochastic S-STDP with the number of learnings for $k=3$ (with $p=0.13$ and $q=0.03$) and $4$ (with $p=0.2$ and $q=0.08$) as well as those using conventional S-STDP ($k=1$ with $p=0.04$ and $q=0.008$) and deterministic S-STDP. The horizontal broken line indicates the 90% accuracy level. (**b)** Comparison of the accuracies achieved in this work with previously reported values^34,44^. (**c)** Relationship between the recognition accuracy and the number of neurons retaining their initially memorised digits after training with 150,000 samples. Data are obtained with various combinations of $k$, $p$ and $q$. The broken line is a visual guide.

In addition to memory maintenance, recognition accuracy is also evaluated to confirm the performance of sigmoidal stochastic S-STDP as a learning rule (Fig. 4a). The recognition accuracy obtained in the cases of stochastic S-STDP ($k=3$ and 4) is apparently higher than that obtained in the conventional case ($k=1$), and is comparable to, or rather slightly better than that obtained in the deterministic case. In fact, we observe that the recognition accuracy in the cases of sigmoidal stochastic S-STDP reaches 90%, which was never achieved with deterministic S-STDP in our simulations. Even higher accuracy is achieved if we use more neurons in the second layer (Fig. 4b). In all the cases of 400, 1,600 and 6,400 neurons, we observe higher accuracy than that reported in the literatures using standard STDP with continuous weights^34^ and conventional stochastic S-STDP with binary weights^44^. In particular, we achieve the accuracy of 97.3% with 6,400 neurons using sigmoidal stochastic S-STDP, exceeding the reported value 95% for standard STDP in the same network^34^ (Table 1).

**Table 1:** Recognition accuracy of MNIST test images using a two-layer SNN.

	Structure	Synapse	STDP	Update	Recognition accuracy (%)
Diehl 2015^34^	784–6400–6400	Continuous	Standard	Deterministic	95
Srinivasan 2019^44^		Binary	Simplified	Stochastic	92.1
This work		Binary	Simplified	Sigmoidal stochastic	97.3

Using sigmoidal stochastic S-STDP as a learning rule, both excellent memory retention and high recognition accuracy are observed simultaneously, indicating the compatibility of memory stability and inference accuracy with this algorithm. In fact, a scatter plot between recognition accuracy and memory maintenance under various conditions shows a clear relationship between the two (Fig. 4c). Such a positive correlation is desirable towards practical use of sigmoidal stochastic S-STDP, because the trade-off between the two would otherwise narrow the opportunities for application of this algorithm in self-learning SNN hardware.

### Effect of variability

One of the inevitable issues in practical use of memristive devices is their variability. Employing stochastic two-level memristors saves us from the variability and uncertainty worries in resistance control, but another concern arises: variability of the probability itself from device to device. Referring to Eq. (3), the device-to-device variability of a SET probability $p$ should be ascribed to that of $\tau$. We assume that the scattering of $\tau$ follows a log-normal distribution because the kinetics of memristive switching phenomena exponentially scales with physical parameters^49,50^. Then, $\tau$ of an arbitrary memristor is given by

8 $$\tau ={\tau }_{0}\mathrm{exp}\left(\sigma r\right),$$

with ${\tau }_{0}$, $\sigma$ and $r$ being a constant, a standard deviation, and a random variable following the standard normal distribution, respectively. We incorporate Eqs. (3) and (8) to perform simulations of learning and recognition with several $\sigma$ values for $k=3$ and $4$.

**Figure 5:**
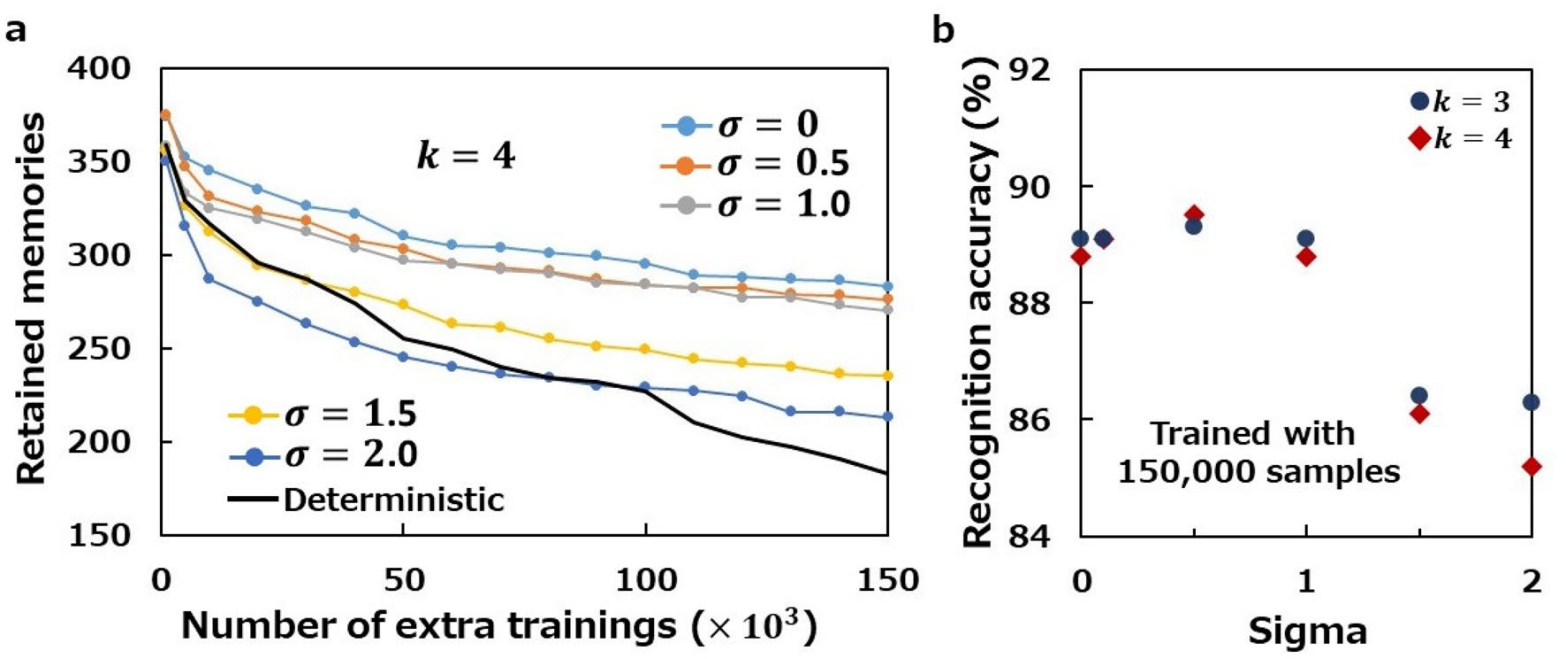
Effect of variability on memory maintenance and recognition accuracy. (**a)** Memory maintenance characteristics obtained with sigmoidal stochastic S-STDP in the case of $k=4$ (with $p=0.2$ and $q=0.08$), with $\sigma$ being varied from 0 to 2.0 as well as that obtained with deterministic S-STDP as a reference (taken from Fig. 1b). **(b)** Recognition accuracy after training with 150,000 samples as a function of $\sigma$ for $k=3$ and $4$.

First, we discuss the memory maintenance characteristics (Fig. 5a). Although degradation in memory maintenance is observed with increasing $\sigma$, the decay rate (i.e., slope) stays unchanged except for the initial stage of the additional learning. In the case of $\sigma \le 1.0$, in particular, sigmoidal stochastic S-STDP always outperforms the deterministic method. With regard to the recognition accuracy, no degradation is observed up to $\sigma =1.0$, followed by a sharp drop at $\sigma =1.5$ (Fig. 5b). To summarise these results, it is reasonable to presume the upper limit of acceptable $\sigma$ to be 1.0.

The literatures show that the variability of SET times in oxide-based memristors ranges by about four orders of magnitude, including both device-to-device and cycle-to-cycle effects^51,52^. With regard to cycle-to-cycle effects, SET times scatter in a range of 50 times in a single memristor exhibiting an exponential SET time distribution^30^. Taking that into account, the contribution of the device-to-device variability to the total scattering range of ${10}^{4}$ is calculated to be ${10}^{4}/50=200$. Assuming that the $3\sigma$ section of the device-to-device distribution covers the range of $200$, we obtain $\sigma =0.88$, which is an acceptable range, as discussed above. The results and discussion in this section convince us that implementing stochastic synapses with multiple-memristor switching devices is a realistic choice.

## Discussion

So far, we have focused on S-STDP. The biggest advantage of this scheme is its simplicity toward hardware implementation. In standard STDP, in contrast, an elaborate mechanism is required to realise exponential-like dependence of the weight update on the spike-timing. Furthermore, synaptic normalisation have to be carried out in every neuron whenever any of the synapses afferent to the neuron is updated^34,40–43^. For synaptic normalisation, a neuron needs to monitor all connected synaptic weights, and when an update occurs, it needs to read them all, sum up the results and divide each weight by the sum. What is more complicated is that division itself does not make sense for a binary weight because a weight can take only 0 or 1. Thus, a highly sophisticated circuitry system would be required to realise synaptic normalisation in hardware. In the scheme of S-STDP, on the other hand, synaptic normalisation is inherent in the algorithm and hence explicit implementation is unnecessary (Supplementary Note 3)^35^. Therefore, it is convenient to employ S-STDP in particular for a binary weight system if it does not underperform standard STDP, which we have shown to be possible by introducing the sigmoidal model.

The concept of a sigmoidal evolution seems to be analogous to the deterministic weight update model proposed by Park^37^. However, we emphasise that the point of our proposal is that it exploits the probabilistic characteristics of random events following a gamma distribution or negative binomial distribution (Supplementary Note 4). To realise a sigmoidal weight evolution in a deterministic fashion, a weight should change depending on its present value, and it may be area-expensive and power-consuming to implement such a mechanism in each synapse. In our proposal, however, there is no need to read the present value or to count $N$, because the potentiation and depression probabilities at the $N$-th trial, ${p}_{k}\left(N\right)$ and ${q}_{k}\left(N\right)$, are inherently $N$-dependent for $k\ge 2$ (see Eq. (3)). All we have to do is to apply constant pulses to a multiple-memristor device for synaptic updates, whatever its state is. This is the benefit of exploiting probabilistic events following a gamma or negative binomial distribution, leading to simplification of the synaptic circuit system.

Although neither elaborate pulsing system nor precise pulse tuning is required to realise sigmoidal stochastic S-STDP, using too many memristors just for a single synapse would be a disadvantage for hardware implementation. To simplify the synaptic circuit, $k$ should be as small as possible. Then a question arises: what is the best $k$? We have already observed excellent learning performance with $k=3$ and $4$. Because no significant difference has been found between the two, we do not expect drastic improvement by increasing $k$ further. In fact, we tried a simulation of $k=5$ with $p=0.29$ and $q=0.12$, and the memory retention and recognition accuracy were very close to those of $k=4$, with $p=0.2$ and $q=0.08$ (parameters used for Fig. 5). Of course, it might be possible to obtain better results by tuning $p$ and $q$ carefully, but that is merely a matter of parameter optimization; we conclude that $k=3$ or $4$ is sufficient.

However, pursuing the optimum $k$ is not meaningless from the viewpoint of practical design. Because the learning speed scales with ${p}^{k}$ or ${q}^{k}$, roughly speaking (see Eqs. (6) and (7)), large $k$ slows down the learning speed. Conversely, large $k$ may be employed if it is convenient to use large $p$ and $q$ for some technical reason. In practical use of memristors, it sometimes happens that, depending on the RESET condition, the simple exponential function of Eq. (3) is no longer valid for a very short pulse^53^. In such a case, it is realistic to apply longer pulses, hence larger $p$ and $q$ with larger $k$, so that Eq. (3), which is the fundamental principle in the theory of this work, can be applied to control the SET probabilities.

Finally, we point that implementation of sigmoidal stochastic S-STDP is possible not only with multiple-memristor switching devices but also with other nano-devices whose stochastic operation is described by gamma distribution (or other probabilistic distribution having a sigmoidal cumulative distribution function), such as magnetic tunnel junction devices^54^, although further studies are necessary to bring that into practice.

## Conclusion

We have proposed sigmoidal stochastic S-STDP with binary synaptic weights, where the probabilities of potentiation and depression depend on the number of repeated trials such that the expected weight $\langle w\rangle$ evolves in a sigmoidal fashion with the potentiation or depression operations, which can be implemented using a pair of switching devices consisting of serially connected multiple binary memristors. As a benchmark test, we performed simulations of MNIST image learning and recognition tasks in two-layer SNNs with binary synapses and showed that learning with the proposed rule outperforms those with deterministic and conventional stochastic S-STDP in memory maintenance and recognition accuracy. Furthermore, we achieved recognition accuracy of 97.3%, exceeding the 95.0% reported for the same two-layer SNN with continuous weights using standard STDP with a synaptic normalisation mechanism. We have also shown that the high performance of sigmoidal stochastic S-STDP holds even if the device-to-device variability of memristors is taken into account. Thus, we conclude that sigmoidal stochastic S-STDP is promising as a local synaptic update rule to be implemented in SNN hardware for unsupervised online learning.

## Methods

### Assignment of memorised digit and derivation of recognition accuracy

In this work, we perform simulations of unsupervised learning and recognition tasks of MNIST images to benchmark the performance of synaptic update rules, following the method of Diehl, et al.^34^. We use a two-layer SNN having 784 input nodes in the first layer and 400 pairs of excitatory and inhibitory neurons in the second layer (in some cases 1600 and 6400 pairs are used, as shown in Fig. 4b). Each input node corresponds to a pixel in the MNIST image, and receives a train of Poisson-distributed spikes whose spiking rate is proportional to the intensity of the pixel. The duration of the spike train is 350 ms per image. The pixel intensity of the MNIST image, which is represented by 256 levels (from 0 to 255), is converted to the spiking rate in Hz by being divided by 4 (i.e. the spike rate ranges from 0 to 63.75 Hz).

The input nodes in the first layer and the excitatory neurons in the second layer are connected in an all-to-all fashion via plastic excitatory synapses, which are updated in accordance with the S-STDP rule. When an excitatory neuron fires, the corresponding inhibitory in the pair neuron provides a lateral inhibition to all the other 399 excitatory neurons, resulting in a winner-take-all function. Note that synaptic connections between excitatory and inhibitory neurons are all non-plastic. The neuron models and hyper-parameters employed in our simulations are also the same as those used by Diehl, et al.^34^, unless otherwise described in the text.

In our simulations, learning and recognition phases are separate; synaptic changes take place only in the learning phase and recognition accuracy is evaluated with fixed synapses. Whereas a subset of sample images out of 60,000 images in the training set are presented to the input nodes for learning, images picked up from the test set are used to evaluate the memory maintenance and recognition accuracy. In the recognition phase, firings of each excitatory neuron are counted during each presentation. A neuron's 'memorised digit' is defined as the label of the image by which the neuron fires most frequently among the presented images. In this way, all the excitatory neurons have their own digit assignments.

To evaluate the memory maintenance, we first train the network with 10,000 samples for initialization and perform a recognition task to assign a memorised digit to each neuron. Then extra training samples are presented to the network for additional learning. After the additional learning, digits are assigned to the neurons again. We count the number of neurons that retain their initial memorised digits as an index of the memory maintenance.

Several neurons can fire during the presentation of an image. Among them, the one that fires most frequently is the representative neuron of the image. If the digit assigned to the representative neuron is equal to the label of the image, the recognition is successful. The recognition accuracy is derived by counting the number of successful recognitions.

### Synaptic update algorithm for sigmoidal stochastic S-STDP

To realise sigmoidal potentiation and depression of $\langle w\rangle$ in the simulation algorithm, we introduce parameters ${m}_{1}, {m}_{2},\cdots ,{m}_{k}$ for potentiation and ${n}_{1}, {n}_{2},\cdots ,{n}_{k}$ for depression, corresponding to the states of the memristors in a pair of multiple-memristor switching devices. The synaptic update runs as Algorithm 1. If a post-synaptic neuron fires under a potentiation condition, $0<{t}_{post}-{t}_{pre}<T$, all ${n}_{1}, {n}_{2},\cdots ,{n}_{k}$ are reset to 0. Then, ${m}_{1}$ is switched to 1 with probability $p$, corresponding to a SET operation of the first memristor, and regardless of whether the switching is successful, the system goes back to standby. In the case where ${m}_{1}$ is already 1, a switch operation is performed for ${m}_{2}$ and the system goes back to standby. If ${m}_{2}$ is also already 1, then we proceed to ${m}_{3}$, and subsequent processing proceeds in the same manner. In this algorithm, we set ${w=m}_{k}$. When ${m}_{k}$ switches to 1, this results in potentiation from $w=0$ to $1$. Otherwise $w=0$ holds. For depression, the algorithm is exactly the same except for ${m}_{i}$ and ${n}_{i}$ being exchanged.
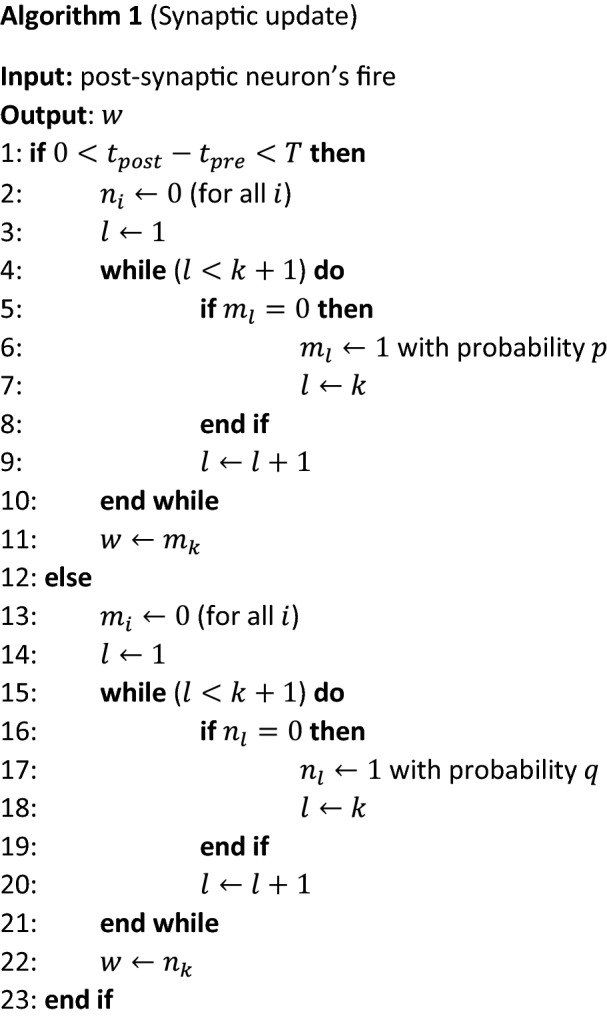


Strictly speaking, this algorithm can reproduce a negative binomial distribution, but not the gamma distribution described by Eqs. (4)–(6). But we can show that a negative binomial is a discrete version of a gamma distribution and can well reproduce the stochastic behaviour of the multiple-memristor switching device (Supplementary Note 4).

## Supplementary Information

Supplementary Information.

## Data Availability

The data that support the findings of this study are available from the authors upon reasonable request.
