# Supplementary material for: Stochastic binary synapses having sigmoidal cumulative distribution functions for unsupervised learning with spike timing-dependent plasticity
Source: Sci Rep. 2021 Sep 14;11:18282. doi: 10.1038/s41598-021-97583-y (PMC8440757; doi:10.1038/s41598-021-97583-y)
Supplement: Supplementary file 1 — Supplementary Information. [file 41598_2021_97583_MOESM1_ESM.pdf]

Supplementary Information

Stochastic binary synapses having sigmoidal cumulative distribution functions for unsupervised learning with spike timing-dependent plasticity

Yoshifumi Nishi*, Kumiko Nomura, Takao Marukame, and Koichi Mizushima
Frontier Research Laboratory, Corporate R&D Center, Toshiba Corporation, 1, Komukai-Toshiba-Cho, Saiwai-ku, Kawasaki, Japan, 212-8582
Email: yoshifumi.nishi@toshiba.co.jp

Supplementary Note 1: Recognition accuracy obtained using conventional stochastic S-STDP

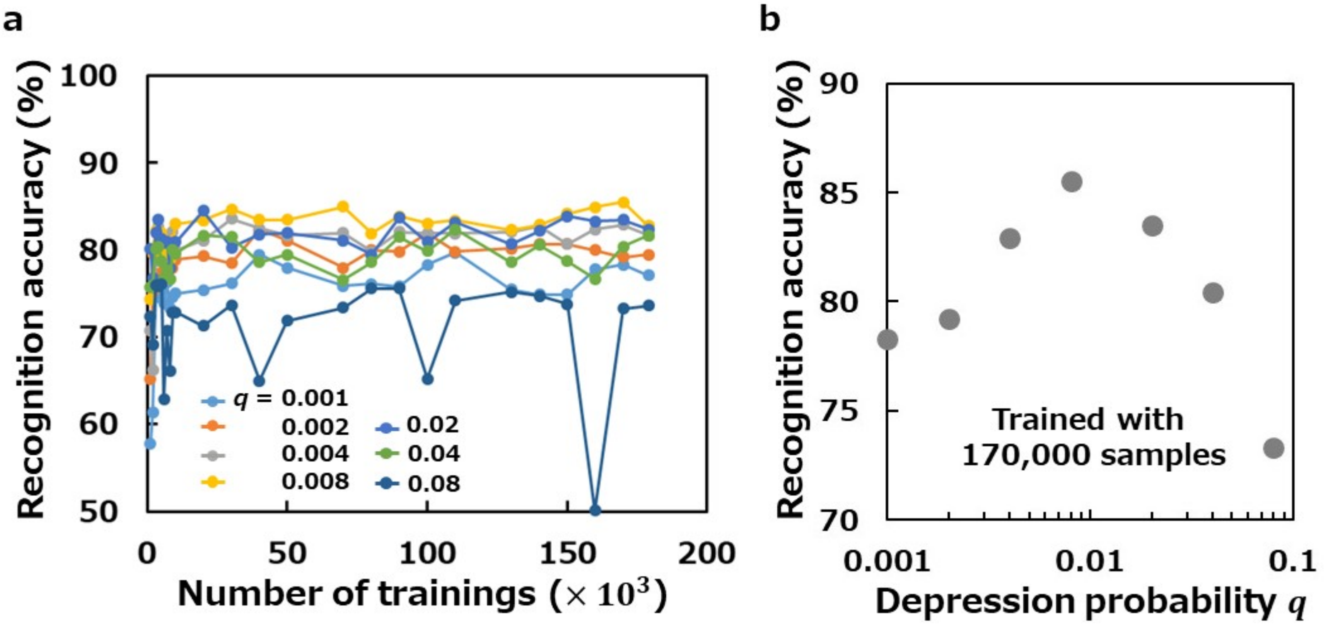

Fig. S1 Recognition accuracy obtained with conventional stochastic S-STDP. **a** Evolution of recognition accuracy as a function of the number of trainings for various q. **b** Recognition accuracy evaluated after 170,000 trainings as a function of q.

The accuracy of MNIST image recognition of a two-layer network trained using conventional stochastic S-STPD is evaluated. While the potentiation probability p is fixed at 0.04, the depression probability q is varied as a parameter. The overall results are shown in Fig. S1a. Starting with $q = 0.001$, the accuracy does not exceed the ceiling of 80 % even if the network is trained with increasingly more samples. The accuracies improve as q increases. The best recognition performance is achieved with $q = 0.008$, and the accuracy reaches 85.5 % after 170,000 trainings. Further increasing q, however, deteriorates the performance. We observe unstable evolution of the recognition accuracy with $q = 0.08$. As can be seen in Fig. S1b, $q \sim 0.008$ gives the maximum accuracy; hence, 85.5% is the highest viable accuracy in this simulation. It is interesting to note that $q = 0.008$ does not necessarily lead to the best memory maintenance (see Fig. 1b), but it is not far from the best.

Supplementary Note 2: Synaptic circuit using multiple memristor-switching devices

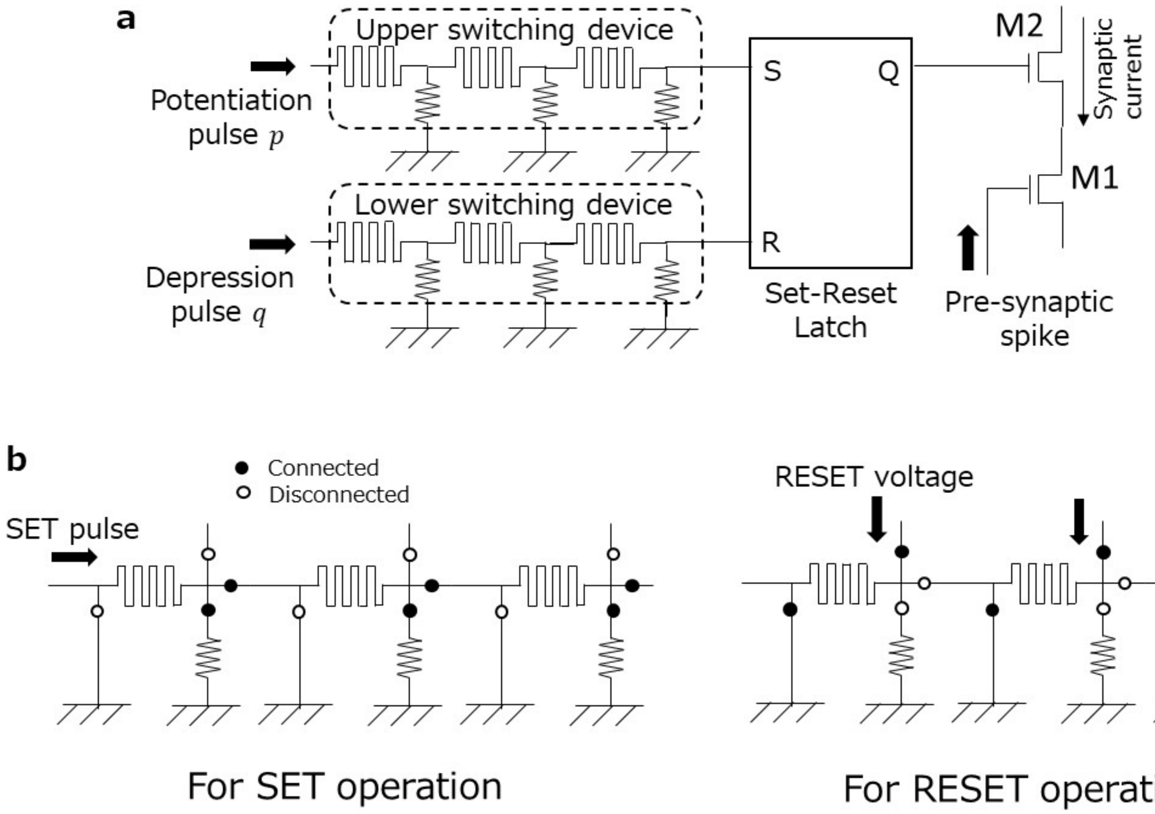

Fig. S2 a Schematics of a synaptic device using a pair of multiple-memristor switches (in the case of $k = 3$). **b** Detailed circuit configurations of a multiple-memristor switch for SET operation (left) and RESET operation (right). Filled and open circles indicate connected and disconnected wires, respectively.

Using a pair of switching devices consisting of serially connected memristors makes it possible to build a stochastic synaptic device having a binary weight. As illustrated in Fig. S2 a, we employ a set-reset (SR) latch, which stores the weight, 0 or 1. The output node of the latch, Q, is connected to switch transistor M2 embedded in a synaptic current circuit, such as a differential pair integrator (DPI) circuit [S1]. If the output from Q is high, M2 is open, and synaptic current flows when a pre-synaptic spike enters transistor M1. This corresponds to $w = 1$. Otherwise, M2 is closed, and synaptic current cannot flow even if a spike opens M1, corresponding to $w = 0$. In this way, the state of the latch represents the weight.

Each input node of the SR latch is connected with a series of memristors via a rectifier. The one connected to the node S is in charge of potentiation and the other controls depression. Assume that all the memristors in the upper switching device are in an HRS, and that the state of the upper node is low (i.e., $w = 0$). If the post-synaptic neuron fires within a period T after the previous pre-synaptic spike, a 'potentiation pulse' is applied to the upper switching device from the left. A potentiation pulse is a voltage pulse that SETs a memristor with probability p. At the same time, all the memristors in the lower device are RESET to an HRS. For RESET operation, the connectivity should be changed as shown in Fig. S2 b. It is no problem if some memristors are already in an HRS. If the potentiation condition is satisfied repeatedly, a series of potentiation pulses is applied to the upper device, inducing SETs in the memristors one after another. Eventually, all the memristors in the upper device are SET to an LRS, and the voltage reaches the node S of the latch. Then, the output from Q turns from low to high, and w is potentiated from 0 to 1. Similarly, if the post-synaptic neuron fires later than T after the previous spike, all the memristors in the upper switching device are RESET and a 'depression pulse' is applied to the lower device from the left. After repeated applications of a depression pulse, all the lower memristors are SET. Then, the voltage reaches the node R of the latch, and the output from Q changes its state from high to low. Accordingly, M2 is closed and w is depressed from 1 to 0.

Note that we do not use RESET operations for stochastic switching. Because, at a glance, a RESET is merely the reverse process of a SET, it seems to be natural to exploit RESETs for depression. However, a RESET is a completely different phenomenon. In fact, whereas a SET occurs as an abrupt jump of the current under voltage application, a RESET is characterised as a gradual decrease of the current [S2]. Whereas a SET is a voltage-driven process with a small leakage current generating Joule heat [S3] and occurs in serially connected memristors one after another, a RESET is a current-driven phenomenon with a large current through the device, making serial RESETs impossible. Once one of the serially connected memristors undergoes a RESET, the current is blocked, and all the other memristors remain in an LRS indefinitely. That is why we use two multiple-memristor devices for potentiation and depression, and apply them as stochastic switches only in one direction from OFF to ON.

As illustrated in Fig. S2 b, we need 4 switches per memristor to change the connectivity for SET and RESET operations, thus at least $4k \times 2 = 8k$ transistors for a pair of the switches. In addition, at least 8 transistors are used for an SR latch. To implement sigmoidal stochastic S-STDP with multiple-memristor switches, we need a chip area for $8(k + 1)$ transistors per synapse. Nevertheless, we believe that the memristive synapse proposed here is still advantageous over a conventional full CMOS synapse with a multi-bit weight, because much more transistors are required for a memory to store the weight value and a decoder to convert the digital weight value to an analogue synaptic current.

Supplementary Note 3: Synaptic normalisation mechanism in S-STDP

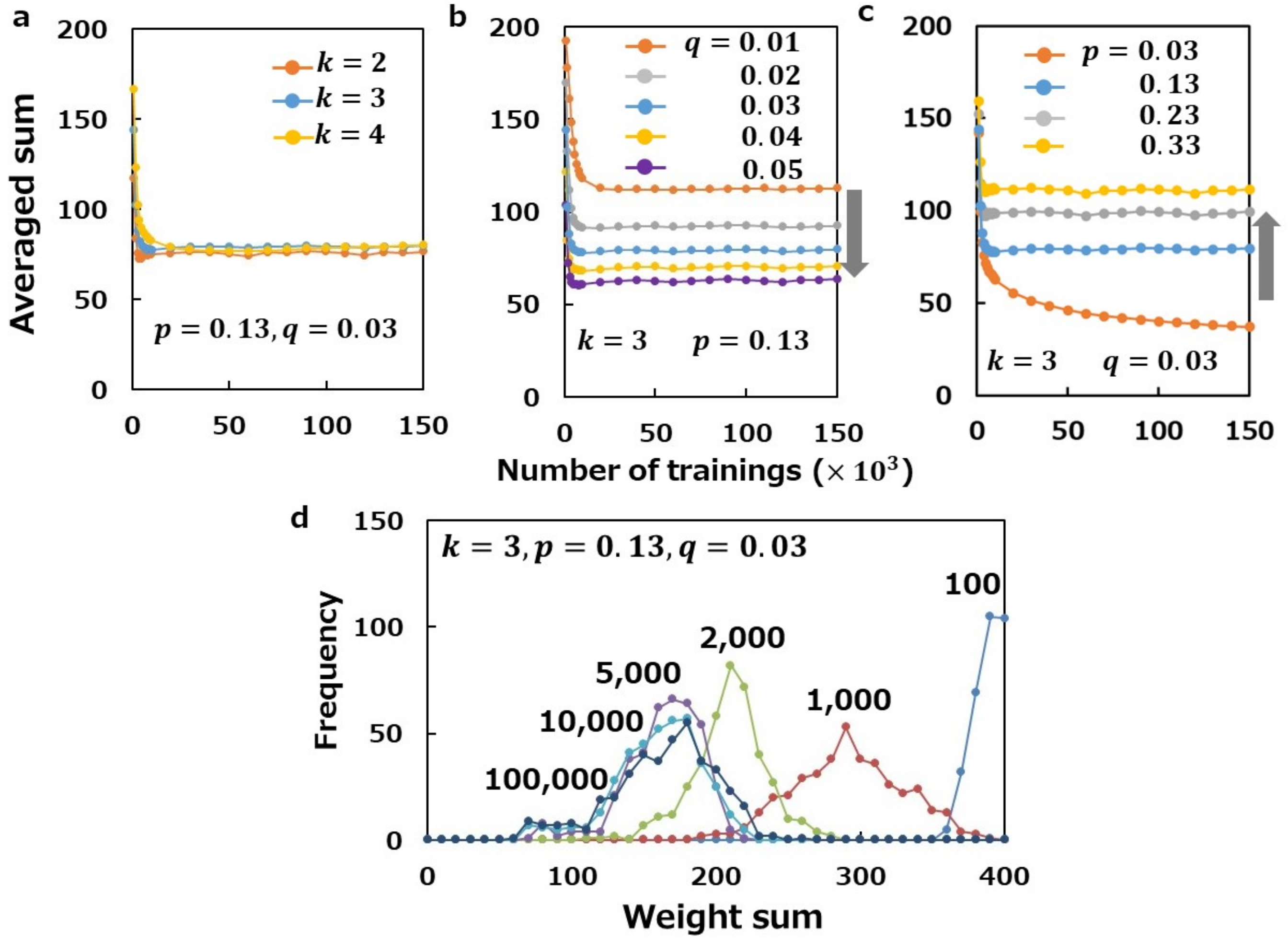

Fig. S3 Synaptic normalisation effect inherent in S-STDP. The sum of the synaptic weights is calculated for each excitatory neuron and averaged over 400 neurons. **a** Variation of k while p and q are kept constant. **b, c** Variation of q or p while the other is kept constant in the case of $k = 3$. **d** Distributions of the weight sums of 400 excitatory neurons after 100, 1,000, 2,000, 5,000, 10,000, and 100,000 trainings in the case of $k = 3$ with $p = 0.13$ and $q = 0.03$.

As mentioned in the Discussion section, synaptic normalisation mechanism is inherent in S-STDP. To confirm this, we calculate the sum of the synaptic weights for each excitatory neuron

$$s_j = \sum_{i=1}^{784} w_{ij}.$$

(S3-1)

Then, among 400 neurons in the first layer, we determine the average

$$S = \sum_{j=1}^{400} s_j \Big/ 400.$$

(S3-2)

The evolution of S with the number of trainings shows that S converges to a constant (around 80) regardless of k, indicating that a mechanism that keeps the weight sum constant is effective (Fig. S3a). The limit of convergence depends on the

combination of p and q. When the depression probability q is increased, S converges to a smaller value because synapses are more likely to be depressed (Fig. S3b). In contrast, the converged value becomes larger if the potentiation probability p increases (Fig. S3c).

Distributions of the weight sums of 400 excitatory neurons are shown in Fig. S3d. After only 100 trainings, the sums are distributed around 400, but with further learning, the distribution shifts to smaller sums. The distribution does not change after training with more than 5,000 samples. This result is not specific to the case of $k = 3$ but also valid for other cases, including $k = 1$, indicating the stability of learning in the S-STDP scheme with binary weights.

Supplementary Note 4: Derivation of a gamma distribution from a negative binomial distribution

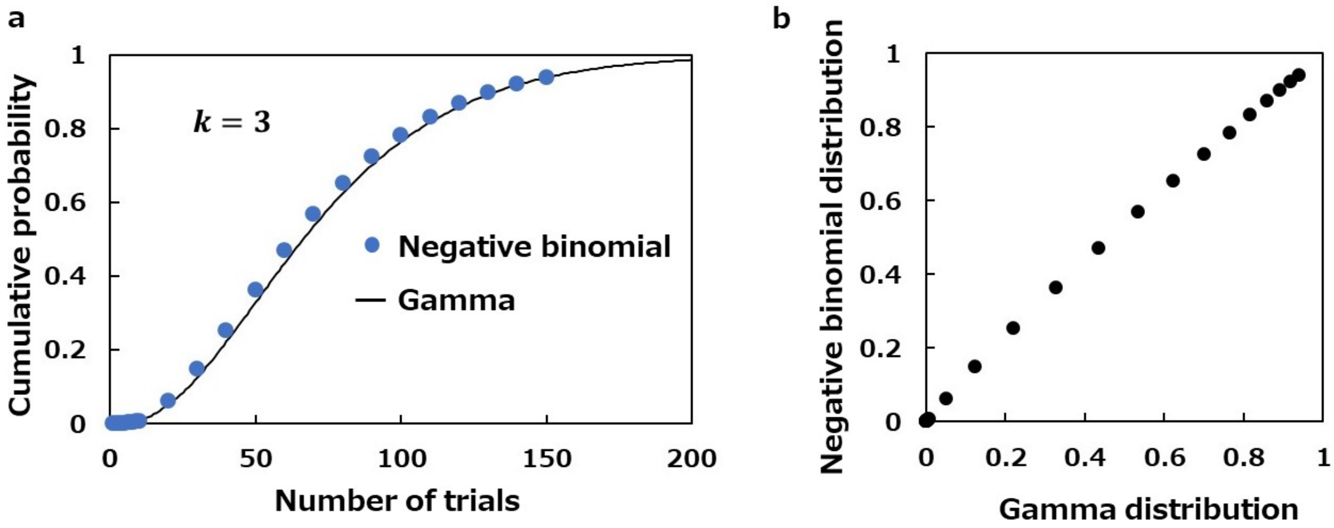

Fig. S4 **a** Cumulative probabilities of negative binomial and gamma distribution with $k = 3$. **b** Quantile-quantile plot.

Let us consider a random event that occurs with a probability p during a trial. The probability that the event occurs for the k-th time during the N-th trial is given by

$$\binom{N-1}{k-1} p^k (1-p)^{N-k}, \tag{S1}$$

known as a negative binomial distribution. If a single pulse applied to a k-memristor switching device can SET at most one memristor (cannot SET two or more), the probability that the switching device switches ON by the N-th pulse is given by equation (S1). In the main text, conversely, we allowed cases where a single pulse can SET more than two memristors; thus, the probability density function is described by equation (4), which describes a gamma distribution.

Equations (4) and (S1) are different at a glance, but we show that equation (4) is a continuous version of equation (S1). Let us consider a negative binomial case. In this case, a single pulse can SET only one memristor. This means that the width of a pulse is the smallest time unit. If we divide the time unit into m subunits, the probability of a SET within a subunit is given by p/m (note that this is because a SET is a purely random event as described by Eq. 3). The probability that the k-th SET occurs at the Nm-th subunit is given by

$$\binom{Nm-1}{k-1}\left(\frac{p}{m}\right)^k\left(1-\frac{p}{m}\right)^{Nm-k} = \frac{1}{(k-1)!}\frac{(Nm-1)!}{(Nm-k)!}\frac{p^k}{m^k}\left(1-\frac{p}{m}\right)^{Nm}\left(1-\frac{p}{m}\right)^{-k}$$
$$\rightarrow \frac{1}{(k-1)!}\frac{N^{k-1}p^k}{m}\exp(-pN), \tag{S2}$$

for $m \rightarrow \infty$. After replacing N with a continuous variable x and $1/m$ with dx, equation (S2) can be written as

$$\frac{x^{k-1}p^k}{\Gamma(k)}\exp(-px)\,dx, \tag{S3}$$

which is the probability density function of the gamma distribution given by equation (4). Figure S4a compares the cumulative probabilities calculated based on negative binomial distribution equation (S1) and on gamma distribution equation (4). The similarity of the two is confirmed also in the quantile-quantile plot shown in Fig. S4b. Excellent coincidence of the two justifies the simulation Algorithm 1 as an operation model of the k-memristor switching device. In addition, this coincidence guarantees that the results and discussions presented in this work are not limited to synapses with multiple-memristor switches, but may be applied to those with other stochastic nano-devices that obey gamma distribution.

References

[S1] Chicca, E., Stefanini, F., Bartolozzi, C. & Indiveri, G., Neuromorphic electronic circuits for building autonomous cognitive systems, Proceedings of the IEEE 102, no. 9, pp.1367-1388 (2014).

[S2] Marchewka, A., et al., Nanoionic resistive switching memories: on the physical nature of the dynamic reset process, Adv. Electron. Mater. 1500233 (2015).

[S3] Nishi, Y., Menzel, S., Fleck, K., Böttger, U. & Waser, R., Origin of the SET kinetics of the resistive switching in tantalum oxide thin films, IEEE Electron Device Lett. vol. 35, no. 2, pp. 259-261 (2014).
